# NR2C2-uORF targeting UCA1-miR-627-5p-NR2C2 feedback loop to regulate the malignant behaviors of glioma cells

**DOI:** 10.1038/s41419-018-1149-x

**Published:** 2018-12-05

**Authors:** Zirong Fan, Jian Zheng, Yixue Xue, Xiaobai Liu, Di Wang, Chunqing Yang, Jun Ma, Libo Liu, Xuelei Ruan, Zhenhua Wang, Yunhui Liu

**Affiliations:** 1 Department of Neurosurgery, Shengjing Hospital of China Medical University, 110004 Shenyang, China; 2 Liaoning Clinical Medical Research Center in Nervous System Disease, 110004 Shenyang, China; 3 Key Laboratory of Neuro-oncology in Liaoning Province, 110004 Shenyang, China; 4 Department of Neurobiology, College of Basic Medicine, China Medical University, 110122 Shenyang, China; 5 Key Laboratory of Cell Biology, Ministry of Public Health of China, China Medical University, 110122 Shenyang, China; 6 Key Laboratory of Medical Cell Biology, Ministry of Education of China, China Medical University, 110122 Shenyang, China; 7 Department of Physiology, College of Basic Medicine, China Medical University, 110122 Shenyang, Liaoning China

## Abstract

Accumulating evidence has highlighted the potential role of non-coding RNAs (ncRNAs) and upstream open-reading frames (uORFs) in the biological behaviors of glioblastoma. Here, we elucidated the function and possible molecular mechanisms of the effect of some ncRNAs and NR2C2-uORF on the biological behaviors of gliomas. Quantitative real-time PCR was conducted to profile the cell expression of lnc-UCA1 and microRNA-627-5p (miR-627-5p) in glioma tissues and cells. Western blot assay was used to determine the expression levels of NR2C2, SPOCK1, and NR2C2-uORF in glioma tissues and cells. Stable knockdown of lnc-UCA1 or overexpression of miR-627-5p in glioma cell lines (U87 and U251) were established to explore the function of lnc-UCA1 and miR-627-5p in glioma cells. Further, Dual luciferase report assay was used to investigate the correlation between lnc-UCA1 and miR-627-5p. Cell Counting Kit-8, transwell assays, and flow cytometry were used to investigate lnc-UCA1 and miR-627-5p function including cell proliferation, migration and invasion, and apoptosis, respectively. ChIP assays were used to ascertain the correlations between NR2C2 and SPOCK1 as well as NR2C2 between lnc-UCA1. This study confirmed that lnc-UCA1 was up-regulated in glioma tissues and cells. UCA1 knockdown inhibited the malignancies of glioma cells by reducing proliferation, migration, and invasion, but inducing apoptosis. We found that lnc-UCA1 acted as miR-627-5p sponge in a sequence-specific manner. Meanwhile, upregulated lnc-UCA1 inhibited miR-627-5p expression. In addition, miR-627-5p targeted 3′UTR of NR2C2 and down-regulated its expression. Moreover, UCA1 knockdown impaired NR2C2 expression by upregulating miR-627-5p. An uORF was identified in mRNA 5'UTR of NR2C2 and overexpression of whom negatively regulated NR2C2 expression. Remarkably, lnc-UCA1 knockdown combined with uORF overepression and NR2C2 knockdown led to severe tumor suppression in vivo. This study demonstrated that the NR2C2-uORF impaired the pivotal roles that UCA1-miR-627-5p-NR2C2 feedback loop had in regulating the malignancies of glioma cells by targeting NR2C2 directly. And this may provide a potential therapeutic strategy for treating glioma.

## Introduction

Glioblastoma multiforme (GBM) is the most common in situ neoplasms in central nervous system which account for 10–15% of all intracranial tumors^1^. Currently, surgery combined with chemotherapy is the main treatment for GBM^2^. However, GBM usually grow aggressively resulting in severe recurrence, and due to its highly invasiveness and insensitivity to chemotherapy, patients usually have poor prognosis, with a median survival of 12–15 months only^3^.

Substantially all genes in human genome are transcribed into RNA, and mostly are noncoding RNAs (ncRNAs)^4^. Primarily, long non-coding RNAs (lncRNAs) and microRNAs (miRNAs) play important roles in the modification and regulation of genes. LncRNAs consist more than 200 nucleotides and modulate gene expression through chromatin remodeling, mRNA degradation, and translation^5,6^. Recently, numerous studies have reported that abnormal expressions of lncRNAs are closely related to malignant behaviors of various tumors including GBM. LncRNA urothelial cancer associated 1 (UCA1) is highly expressed in a variety of tumor cells and leads to poor prognosis^7^, such as bladder cancer^8^ and oral squamous cell carcinoma^9^. But the impact that UCA1 may have on glioma remained unclear. MiRNAs bind to 3'untranslated region (3'UTR) of mRNAs of target genes^10^, resulting in the degradation of mRNAs or the suppression of translation process^11,12^. Plenty of researches have reported the involvement of miRNAs in regulating tumors malignancies^13^. Recent researches have shown that miR-627, which is a possible target of UCA1, expressed significantly low in several tumors including colorectal cancer^14^. However, the potential role of miR-627-5p in human gliomas remained unclear.

Transcription factor nuclear receptor subfamily 2 group C member 2 (NR2C2) belongs to the nuclear hormone receptor family and functions in many biological processes, such as development and homeostasis^15,16^. We predicted possible binding sites of miR-627-5p in NR2C2 mRNA. Large scale of studies have shown that NR2C2 played an important role in the development of tumor, such as lung cancer and prostate cancer^17,18^. But the role of NR2C2 in gliomas has not been clearly reported yet.

Upstream open-reading frames (uORFs) are major regulatory elements that exist in eukaryotic mRNAs 5'UTR, which play crucial roles in the process of gene expression^19^, usually start with the uAUG codon and end with the stop codon^20^. By preventing ribosomes from acting on the major initiation site and inhibiting the translation of mRNA, uORFs are involved in the translational process of proteins^21,22^. Genetic and bioinformatic studies suggested that lacking uORFs may lead to diseases^23–26^. Using ORF Finder, we predicted an uORF in the 5'UTR of NR2C2 mRNA variant 1. And we are about to clearify its roles in regulating NR2C2 and UCA1/miR-627-5p/NR2C2 pathway.

In this study, we first examined the expression levels of uORF, UCA1, miR-627-5p, and NR2C2 in glioma tissues and cell lines. Based on these results, the interaction among UCA1, miR-627-5p, and NR2C2 in regulating malignant behaviors of gliomas, as well as the role of NR2C2-uORF in this pathway were also explored.

## Materials and methods

### Clinical specimens

All glioma samples and normal human brain tissues were acquired from the Department of Neurosurgery in Shengjing Hospital of China Medical University. All patients are well informed of the usage of tissues and had signed an informed consent form before the surgery was performed and all patients got approved from the Institutional Research Ethics Committee of Shengjing Hosipital. All specimens were immediately placed well in liquid nitrogen after surgery and divided into two groups: Grade I–II (*n* = 5), Grade III–IV (*n* = 5) according to the WHO classification of tumors in the central nervous system (2007). Normal human brain tissues (*n* = 5) were obtained from brain trauma cases and these were used as negative controls.

### Cell culture

Human U87 and U251 glioma cell lines were purchased from Shanghai Institutes for Biological Sciences Cell Resource Center. U87 cells were cultured in high glucose Dulbecco’s modified Eagle medium (DMEM, Gibco, NY, USA) added with 10% fetal bovine serum (FBS, Gibco, CA, USA). U251 cells were cultured in DMEM: Nutrient Mixture F-12 (DMEM/F-12, Gibco, NY, USA) added with 10% FBS (Gibco, CA, USA). Normal human astrocytes (NHAs) were purchased from the Sciencell Research Laboratories (Carlsbad, CA, USA) and cultured in astrocyte medium (Carlsbad, CA, USA). All cells were incubated at a humidified incubator with the temperature of 37 °C and 5% CO_2_.

### Quantitative RT-PCR assay

Total RNA was extracted from tissues and cells by using Trizol reagent (Invitrogen, Carlsbad, CA, USA) according to the protocol of the manufacturer. RNA concentrations were detected by 260/280 nm absorbance using the Nano-drop spectrophotometer (ND-100, Thermo, Waltham, MA). SYBR Premix Ex Taq and TaqMan gene expression assays (Applied Biosystems, Foster City, CA, USA) were used to detect expression levels of lnc-UCA1, NR2C2, and GAPDH. TaqMan MicroRNA Reverse Transcription kit and Taqman Universal Master Mix II (Applied Biosystems) were used to detect miR-627-5p and U6 expression. Relative expression values were normalized and calculated to represent fold change in gene expression using relative quantification as 2^−△△CT^.

### Cell transfection

The short hairpin RNAs against human UCA1 (NR_015379.3) gene were designed and constructed in a pGPU6/Neo vector (UCA1 (−); GenePharma, China), and their empty vectosr were constructed and used as the negative control (NC) (UCA1(−) NC). Full-length NR2C2 was ligated into a pGCMV/MCS/Neo vector (NR2C2(+)), and its empty vector was constructed and used as negative control (NR2C2(+)NC). The short hairpin RNAs of NR2C2 (NM_003298.4) were constructed into a pGPU6/Neo vector (NR2C2(−); GenePharma, China), and their empty vectors were constructed and used as the negative controls (NR2C2(−)NC; GenePharma, China). Both U87 and U251 cells were seeded in 24-well plates and transfected using reagent (Lipo3000, Life Technologies) and Opti-MEM I (Life Technologies) when the confluence reached 80% approximately. According to the manufacturer’s instructions, vectors were used at a concentration of 500 ng/µl. G418 (Invitrogen, Carlsbad, CA) was used to select stable cell lines and G418-resistant clones were obtained after 24 days. Transfection efficacy was evaluated using qRT-PCR assay. AgomiR-627-5p, antagomiR-627-5p, and their negative control sequences (agomiR-627-5pNC and antagomiR-627-5pNC, GenePharma) were transiently transfected into glioma cells to evaluate the effects of miR-627-5p. And they were also transiently transfected into glioma cells that were stably transfected with UCA1 or NR2C2 plasmids. Transiently transfected cells were harvested 48 h after transfection.

### Cell proliferation assay

Glioma cells were seeded at a density of 2000 cells per well in 96-well plates and assayed 48 h after transfection. According to the protocol of the manufactuer, 10 μl CCK8 (CCK-8, Dojin, Japan) solution was added into each well, all cells were incubated for another 2 h at 37 °C. Absorbance was measured and recorded at 450 nm.

### Cell migration and invasion assay

Cell migration and invasion abilities were measured using the 24-well transwell chambers with 8 μm polycarbonate membrane (Corning, NY, USA). Filter was first pre-coated with 500 ng/ml matrigel solution for cell migration assay (BD, Franklin Lakes, NY, USA) and incubated for 4 h at 37 °C, then 500 μl of 10% FBS medium was placed in the lower chamber, and 100 μl serum-free medium was placed in the upper chamber. After cells were incubated at 37 °C for 14–24 h, cells on the upper membrane surface were scraped off. The cell invasion assay is almost the same to the migration assay and the only difference is that the filter do not need to be pre-coated with 500 ng/ml matrigel solution. Surface were fixed with mixture of methanol and glacial acetic acid at the ratio of 3:1 for 30 min and then dried before they were stained with 15% giemsa solution for 6–8 h. Then stained cells were observed and counted using an inverted microscope and an average number within five randomly chosen fields was obtained.

### Quantification of apoptosis by flow cytometry assay

Apoptosis ability was detected after cells were stained with Annexin V-APC/7-AAD (BD Biosciences) according to the manufacturer’s instructions. Cells were resuspended in 1× binding buffer at a concentration of 1 × 10^6^ cells/ml after washing three times with cold phosphate-buffered saline. Cells were incubated for another 15 min at room temperature in a dark case after 5 μl allophycocyanin (APC) and 5 μl 7-aminoactinomycin D (AAD) were added, and the total volume was made up to 500 μl using 1× binding buffer. Cell samples were then analyzed and counted by flow cytometry (FACScan, BD Biosciences).

### Western blot analysis

Total protein of glioma cells were lysed using RIPA (Beyotime Institute of Biotechnology) buffer supplemented with protease inhibitors in icy water and centrifuged at 17,000 × *g* 4 °C for 35 min. Equal amount of protein samples (40 ng) were electrophoresed in SDS–polyacrylamide gel electrophoresis (SDS–PAGE) and then transferred to PVDF membranes. Membranes were then incubated in 5% non-fat milk for 2 h cosisting of Tris-buffered saline (TBS) and 0.1% Tween-20 at room temperature in order to block non-specific bindings. The protein then went immunoblotting against NR2C2 (1:500, Santa Cruz Biotechnology, Santa Cruz, USA), SPOCK1(1:500, Abcam, Cambridge, MA), NR2C2-uORF (1:1000, Beijing Protein Innovation, China), and GAPDH (1:5000, Proteintech, Chicago, IL, USA). The membranes were washed in 1× TBS for three times and then incubated with secondary antibodies (goat anti-rabbit or goat anti-mouse, 1:10,000; Proteintech, Chicago, IL, USA) at room temperature for 2 h. After washing in 1× TBS for three times, immune complex were visualized by enhanced chemiluminescence (ECL kit, Beyotime Institute of Biotechnology, Jiangsu, China) and scanned using ChemImager 5500 V2.03 software. The integrated density values (IDV) of all bands were analvzed using FluorChem 2.0 software.

### Luciferase reporter assays

Sequences of potential binding sites of miR-627-5p in UCA1 full length and NR2C2 3'-UTR sequences and their mutant sequences were amplified by PCR and then cloned into a pmirGLO dual-luciferase Vector (Promega, Madison, WI, USA) to construct luciferase reporter vector (UCA1-Wt and NR2C2-Wt; GenePharma). Glioma cells were seeded in 96-well plates and the cells were co-transfected with UCA1-Wt (or UCA1-Mut) or NR2C2-Wt (or NR2C2-Mut) and agomiR-627-5p or agomiR-627-5p-NC plasmid when they reached about 80% confluence. The luciferase activities were measured 48 h after transfection by Dual-Luciferase reporter assay kit (Promega, USA).

### Chromatin immunoprecipitation assay

Chromatin immunoprecipitation assay was conducted using the Simple Chip Enzymatic Chromatin IP kit (Cell Signaling Technology, Danvers, MA, USA) based on the protocol of manufacturer. Briefly, cells were first cross-linked with 1% formaldehyde for 10 min and then quenched after adding glycine. In order that chromatin was split, cells were collected in lysis buffer containing 1% phenylmethanesulfonyl fluoride (PMSF) and then digested by micrococcal nuclease. Lysate at the concentration of 2% was used as an input reference. The samples was incubated with 5 μg of anti-NR2C2 antibody (Santa Cruz, LA, USA) or normal rabbit IgG for later reaction.Then samples with antibodies were treated with Protein G Agarose Beads and an overnight incubation at 4 °C with gentle shaking. The DNA crosslink samples was reversed in 5 mol/l NaCl and Proteinase K at 65 °C for 2 h and then purified DNA samples was obtained. Immunoprecipitated DNA was then amplified by PCR using their specific primers.

### Tumor xenografts in nude mice

Cells with stable expression of UCA1(−), NR2C2(−), uORF(+) were used for the in vivo study. The uORF overexpression plasmid was transfected into cells stable co-transfected with UCA1(−) and NR2C2(−) and then cells were selected using G418 until stable expression of UCA1(−)+uORF(+)+NR2C2(−). Nude mice for experiments were divided into five groups: control, sh-UCA1, uORF(+), NR2C2(−), and UCA1(−)+uORF(+)+NR2C2(−). Four-week-old nude BALB/c athymic mice were purchased from the Cancer Institute of the Chinese Academy of Medical Science in Beijing. All procedures were conducted according to the Care and Use of Laboratory Animals, and approvals from the Experiment Animal Care Committee of Shengjing Hospital were obtained. Each nude mouse was injected in the right flank area with 3 × 10^5^ cells. Tumor volume and weight were measured every 5 days. All tumors were harvested and weighed at the end of the 7th week. Intracranial experiments were performed by injecting 3 × 10^5^ cells to nude mice into thier right striatum. Numbers of surviving mice was recorded every 5 days, and data was performed and analyzed using a Kaplan–Meier survival curve.

### Statistical analysis

Data were presented as means ± s.d. from at least three experiments independently. GraphPad Prism v5.01 (GraphPad Software, CA, USA) with the Student’s *t*-test (two-tailed) or one-way ANOVA was used for statistical analysis. Differences were considered to be significant when *P* < 0.05.

## Results

### UCA1 acted as an oncogene in glioma tissues and cells

**Fig. 1:**
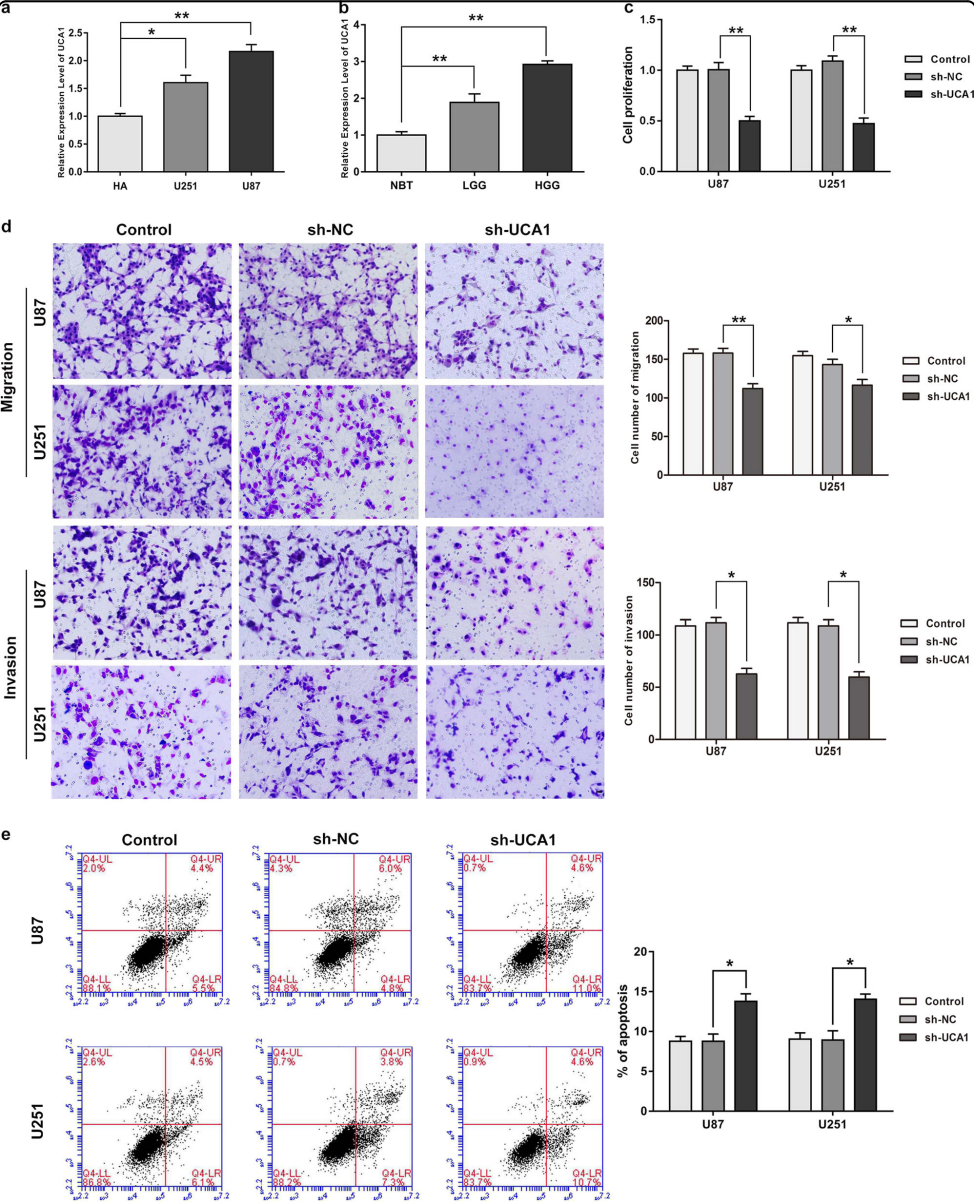
UCA1 acted as an oncogene in human glioma tissues and cells. **a** The expression of UCA1 in human normal astrocytes and glioblastoma multiforme (GBM) cell lines (U87 and U251). Error bars represent as the mean ± SD (*n* = 5, each group). *P < 0.05, **P < 0.01. **b** The expression of UCA1 in normal brain tissues (NBTs), Grade I–II glioma tissues (LGGs), Grade III–IV glioma tissues (HGGs). Error bars represent as the mean ± SD (*n* = 5, each group). **P < 0.01. **c** Effect of UCA1 knockdown on cell proliferation of U87 and U251 cells. **d** Effect of UCA1 knockdown on cell migration and invasion of U87 and U251 cells. **e** Effect of UCA1 knockdown on cell apoptosis of U87 and U251 cells. Error bars represent as the mean ± SD (*n* = 3, each group). *P < 0.05. Scalebars represent 20 μm

qRT-PCR assay was conducted to assess UCA1 expression levels in normal brain tissues (NBTs), low-grade glioma tissues (grade I–II), high-grade glioma tissues (grade III–IV), as well as in NHAs and U87 and U251 glioma cells. Compared with the NHA group, expression of UCA1 was signifcantly up-regulated in U87 and U251 cells, as shown in Fig. 1a (*P* < 0.05). Figure 1b shows that compared with NBTs, UCA1 expression was promoted in human glioma tissues (*P* < 0.05).

### Knockdown of UCA1 impaired malignancies in glioma cells

To assess the functional role of UCA1 in glioma cells, stable UCA1-silenced constructs were used. The transfection efficiency after 72 h is showed in Supplementary Figure 1a (*P* < 0.05). As shown in Fig. 1c, Cell Counting Kit-8 (CCK8) assay revealed that compared with NC group, a decrease occurred in proliferation ability of glioma cells in the UCA1(−) group (*P* < 0.05). A similar tendency occurred in transwell assay (Fig. 1d, *P* < 0.05). On the contrary, cell apoptosis rates were higher in the UCA1(−) group (Fig. 1e, *P* < 0.05).

### MiR-627-5p acted as a tumor suppressor in glioma cells

**Fig. 2:**
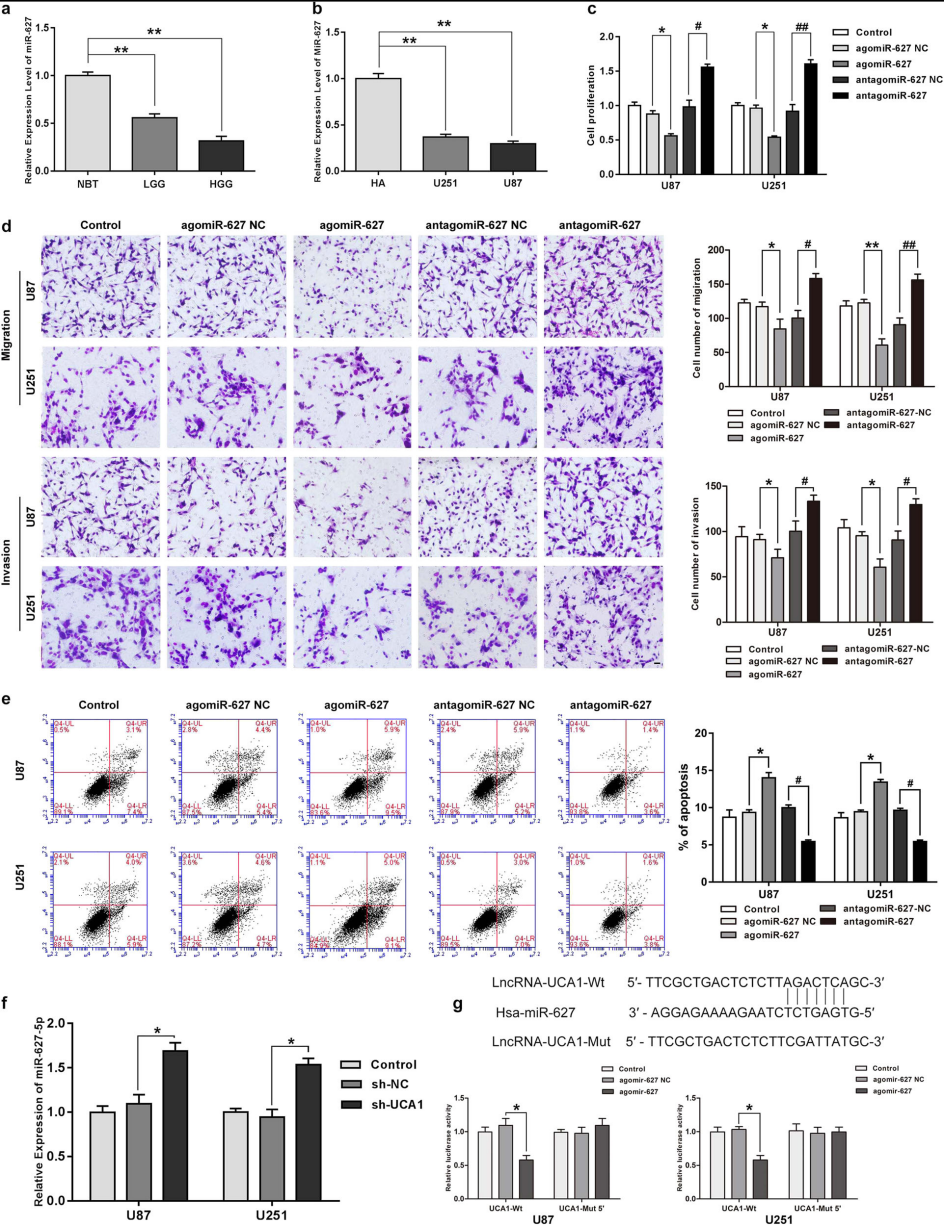
MiR-627-5p acted as a tumor suppressor in glioma cells and was down-regulated by UCA1. **a** MiR-627-5p expression in normal brain tissues (NBTs), Grade I–II glioma tissues, Grade III–IV glioma tissues. Error bars represent as the mean ± SD (*n* = 5, each group). **P < 0.01, ^##^*P* < 0.01. **b** MiR-627-5p expression in human normal astrocytes and GBM cell lines (U87 and U251). Error bars represent as the mean ± SD (*n* = 5, each group). **P < 0.01, ^##^*P* < 0.01. **c** Effect of miR-627-5p on cell proliferation of U87 and U251 cells. **d** Effect of miR-627-5p on cell migration and invasion of U87 and U251 cells. **e** Effect of miR-627-5p on cell apoptosis of U87 and U251 cells. Error bars represent as the mean ± SD (*n* = 3, each group). *P < 0.05, ^#^*P* < 0.05. Scale bars represent 20 μm. **f** Relative expression of miR-627-5p after cells transfected with the expression of UCA1 changed. Error bars represent as the mean ± SD (*n* = 3, each group). *P < 0.05. **g** Relative luciferase activity was performed by dual-luciferase reporter assay. Error bars represent as the mean ± SD (*n* = 3, each group). *P < 0.05. Scalebars represent 20 μm

MiR-627-5p expression was detected by qRT-PCR. Results in Fig. 2a and b indicates that compared with NBTs and NHAs, miR-627-5p expression was low in glioma tissues and cells, respectively(*P* < 0.05). For investigating further the role of miR-627-5p in glioma cells, agomir-627-5p and antagomir-627-5p were transfected into human glioma cells. The efficiency of transfection after 72 h is shown in Supplementary Figure 1b (*P* < 0.05). Overexpression of miR-627-5p resulted in decreased cell proliferation, migration, and invasion compared with NC group (*P* < 0.05), but induced apoptosis of glioma cells significantly (*P* < 0.05), while inhibition of miR-627-5p expression led to promotion of cell proliferation, migration, and invasion, but led to an inhibiton in cell apoptosis (Fig. 2c–e, *P* < 0.05).

### UCA1 targeted miR-627-5p and regulated miR-672-5p negatively

**Fig. 3:**
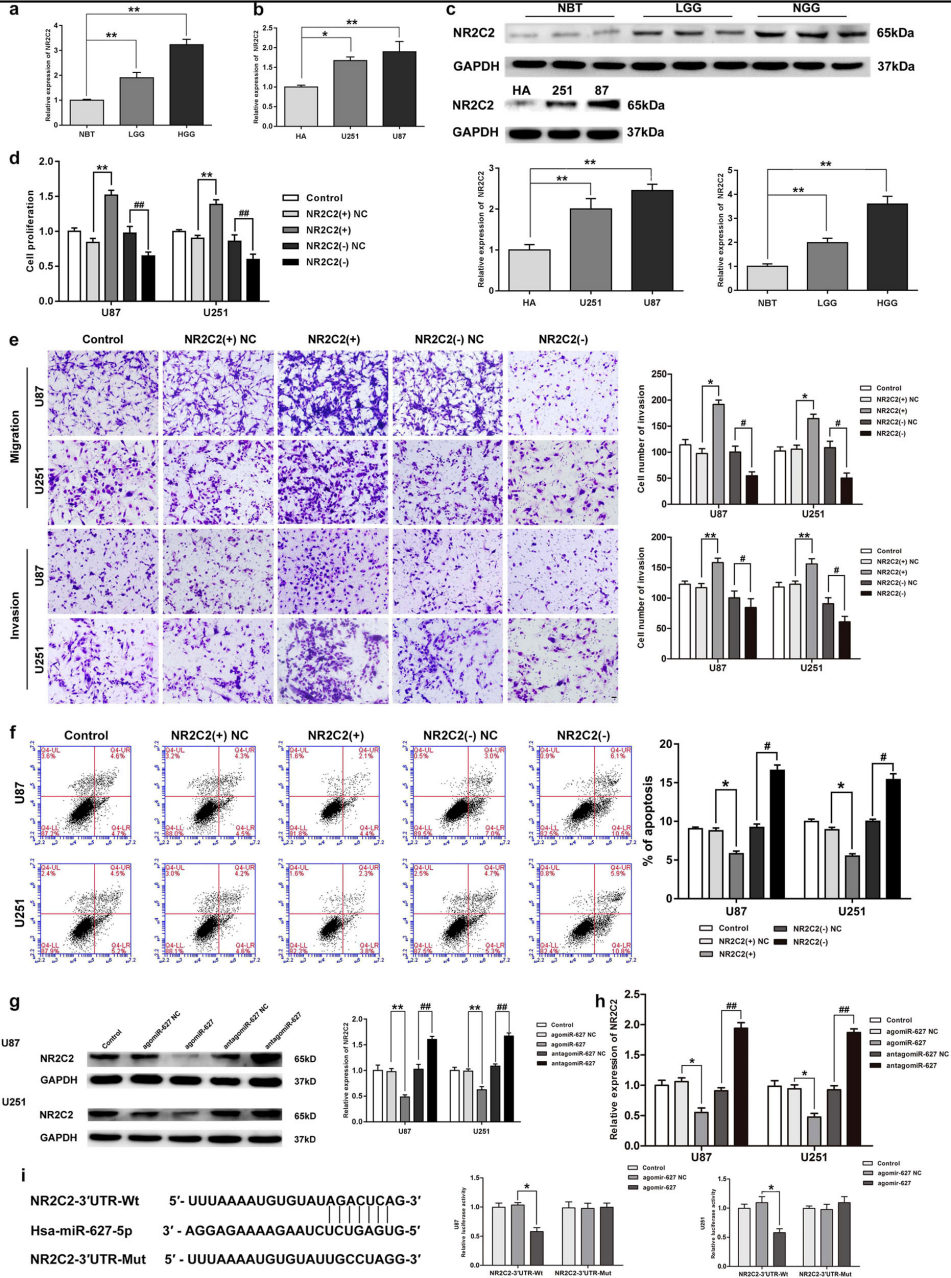
NR2C2 acted as an oncogene in glioma cell lines and was inhibited by miR-627-5p. **a** The expression of NR2C2 in normal brain tissues (NBTs), low grade (grade I–II) glioma tissues (LGGs), high grade (grade III–IV) glioma tissues (HGGs). Error bars represent as the mean ± SD (*n* = 5, each group). **P < 0.01. **b** NR2C2 expression in human normal astrocytes and glioblastoma multiforme (GBM) cell lines (U87 and U251). Error bars represent as the mean ± SD (*n* = 5, each group). *P < 0.05, **P < 0.01. **c** The protein expression of NR2C2 in human glioma tissues and glioma cell lines. Error bars represent as the mean ± SD (*n* = 3 for each tissue groups, *n* = 3 for each cell groups). **P < 0.01. **d** Effect of NR2C2 on cell proliferation of U87 and U251 cells. **e** Effect of NR2C2 on cell migration and invasion of U87 and U251 cells. **f** Effect of NR2C2 on cell apoptosis of U87 and U251 cells. Error bars represent as the mean ± SD (*n* = 3, each group). *P < 0.05, ^#^*P* < 0.05. Scale bars represent 20 μm. **g** Western blot analysis for NR2C2 in U87 and U251 cells, after cells were transfected with the expression of miR-627-5p changed. Error bars represent as the mean ± SD (*n* = 3, each group). **P < 0.01, ^##^*P* < 0.01. **h** Relative expression of NR2C2 after cells transfected with the expression of miR-627-5p changed. Error bars represent as the mean ± SD (*n* = 3, each group). *P < 0.05, ^##^*P* < 0.01. **i** Relative luciferase activity was performed by dual-luciferase reporter assay. Data represent mean ± SD (*n* = 3, each). *P < 0.05. Scalebars represent 20 μm

A possible miR-627-5p-binding site in UCA1 was predicted by a bioinformatics databases (miRanda). Figure 3f shows that compared with NC group, knockdown of UCA1 increased miR-627-5p expression (*P* < 0.05). We then cloned reporter plasmids containing the predicted miR-627-5p-binding site (UCA1-Wt and UCA1-Mut) and dual-luciferase reporter assay was used to investigate whether miR-627-5p bound to UCA1 functionally. Cotransfection of agomir-627-5p and UCA1-Wt significantly decreased luciferase activity as shown in Fig. 3g (*P* < 0.05), while no changes occurred in cotransfection of NC and UCA1-Wt, neither in cotransfection of agomir-627-5p and UCA1-Mut. These results suggested that the miR-627-5p-binding sites within UCA1 worked functionally.

### NR2C2 promoted malignant behaviors in glioma cell lines

RNA expression of NR2C2 in glioma tissues and cells were evaluated by qRT-PCR assay. As shown in Fig. 3a, b, significant elevation of NR2C2 was observed in glioma tissues and cell lines compared with NBTs and NHA, respectively (*P* < 0.05). Moreover, NR2C2 protein expression level was up-regulated in glioma cells compared with NHAs (*P* < 0.05), and also in glioma tissues compared with NBTs (Fig. 3c, *P* < 0.05). We explored the possible functional roles of NR2C2 in glioma cells after they were transfected with stable silenced and overexpressed constructs. The efficiency of transfection after 72 h was displayed in Supplementary Figure 1c (*P* < 0.05). CCK-8 assay was conducted and revealed the increase of proliferation of glioma cells in NR2C2(+) group (*P* < 0.01), and the decrease in NR2C2(−) group compared with the NC group (Fig. 3d, *P* < 0.01). As shown in Fig. 3e, numbers of cells in migration and invasion increased significantly in NR2C2(+) group compared with the NC group (*P* < 0.05) while opposite results occurred in the NR2C2(−) group (*P* < 0.05). Up-regulated NR2C2 impaired apoptosis ability of glioma cells significantly compared with NC group (Fig. 3f, *P* < 0.05), while NR2C2 knockdown showed the opposite result (*P* < 0.05).

### MiR-627-5p inhibited NR2C2 expression

Using the bioinformatics database TargetScan, we predicted that the 3′UTR of NR2C2 contained a putative-binding site of miR-627-5p. Western blot assay was conducted to assess whether miR-627-5p could regulate NR2C2 negatively. Figure 3g shows that compared with NC group, NR2C2 expression was inhibited in agomir-627-5p group (*P* < 0.01), but increased in antagomir-627-5p group(*P* < 0.01). RNA expression level showed the same results (Fig. 3h, *P* < 0.05). Subsequently, we then cloned reporter plasmids containing the wide type 3′UTR of NR2C2 (NR2C2-3′UTR-Wt) and mutant type 3′UTR of NR2C2 (NR2C2-3′UTR-Mut) to testify whether NR2C2 acted as a functional target of miR-627-5p. Figure 3i shows that cotransfection of NR2C2-3′-UTR-Wt and agomir-627-5p decreased the luciferase activity significantly, while cotransfection of NR2C2-3′-UTR-Wt and agomir-627-5p NC did not change the luciferase activity. Cotransfection of agomir-627-5p and NR2C2-3′-UTR-Mut group showed no change in the luciferase activity either. These findings indicated that putative binding site of miR-627-5p in the3′UTR of NR2C2 was functional.

### NR2C2-uORF regulated NR2C2 expression negatively

**Fig. 4:**
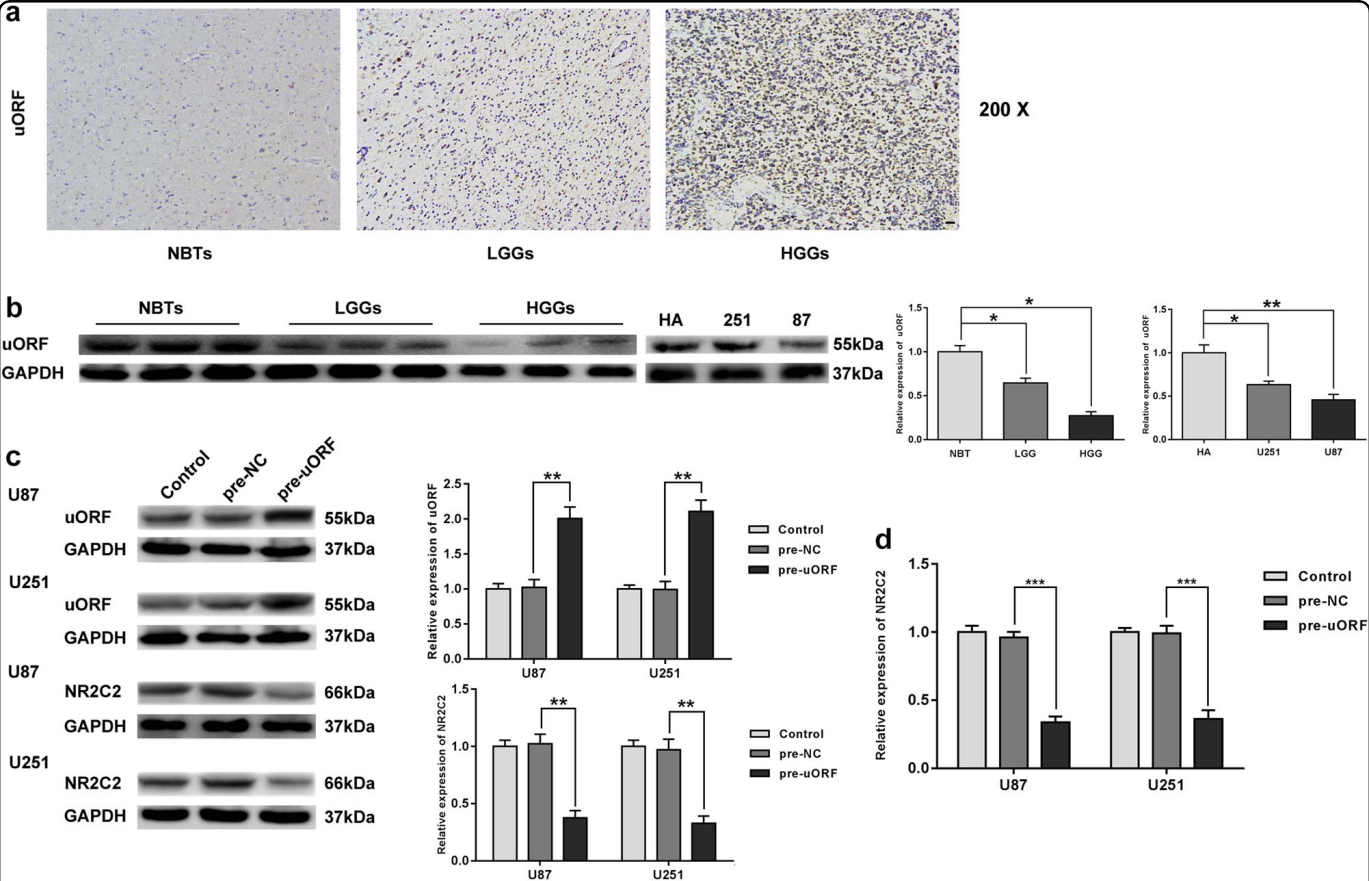
NR2C2-uORF-mediated NR2C2 expression by down regulating its mRNA. **a** Immunohistochemistry of NR2C2-uORF peptide in nontumorous brain, low-grade glioma, and high-grade glioma tissues. Original magnification: ×100. Scale bar = 50 μm. **b** The expression of uORF in normal brain tissues (NBTs), low grade (grade I–II) glioma tissues (LGGs), and high grade (grade III– IV) glioma tissues (HGGs). Error bars represent as the mean ± SD (*n* = 3, each group). *P < 0.05, **P < 0.01. **c** Western blot analysis for uORF and NR2C2 in U87 and U251 cells, after uORF was overexpressed in cell lines. Error bars represent as the mean ± SD (*n* = 3, each group). **P < 0.01. **d** Relative expression of NR2C2 mRNA variant 1 in U87 and U251 cells with the overexpression of uORF. Error bars represent as the mean ± SD (*n* = 3, each group). ***P < 0.001

Immunohistochemistry analysis showed that NR2C2-uORF located in the nucleus and it positively correlated with the progression of glioma pathological grades (Fig. 4a). The expression level of NR2C2-uORF was measured by Western blot as displayed in Fig. 4b, uORF expression was impaired in human glioma tissues compared with that in NBTs (*P* < 0.05), which was negatively correlated with the pathological grade of glioma tissues, and the expression level of uORF in NHA was higher than that in glioma cells (*P* < 0.05). To explore the effect of uORF on NR2C2, we transfected glioma cells with pre-uORF and pre-NC constructs and examined uORF and NR2C2 expression. As shown in Fig. 4c, compared with NC group, pre-uORF group showed a significant increase in uORF (*P* < 0.01) along with a sharp decrease in NR2C2 (*P* < 0.01). We then measured the expression level of NR2C2 mRNA variant 1 in glioma cells. The expression level of NR2C2 mRNA decreased in pre-uORF group compared with pre-NC group (*P* < 0.001).

### UCA1 knockdown inhibited NR2C2 expression by targeting miR-627-5p

**Fig. 5:**
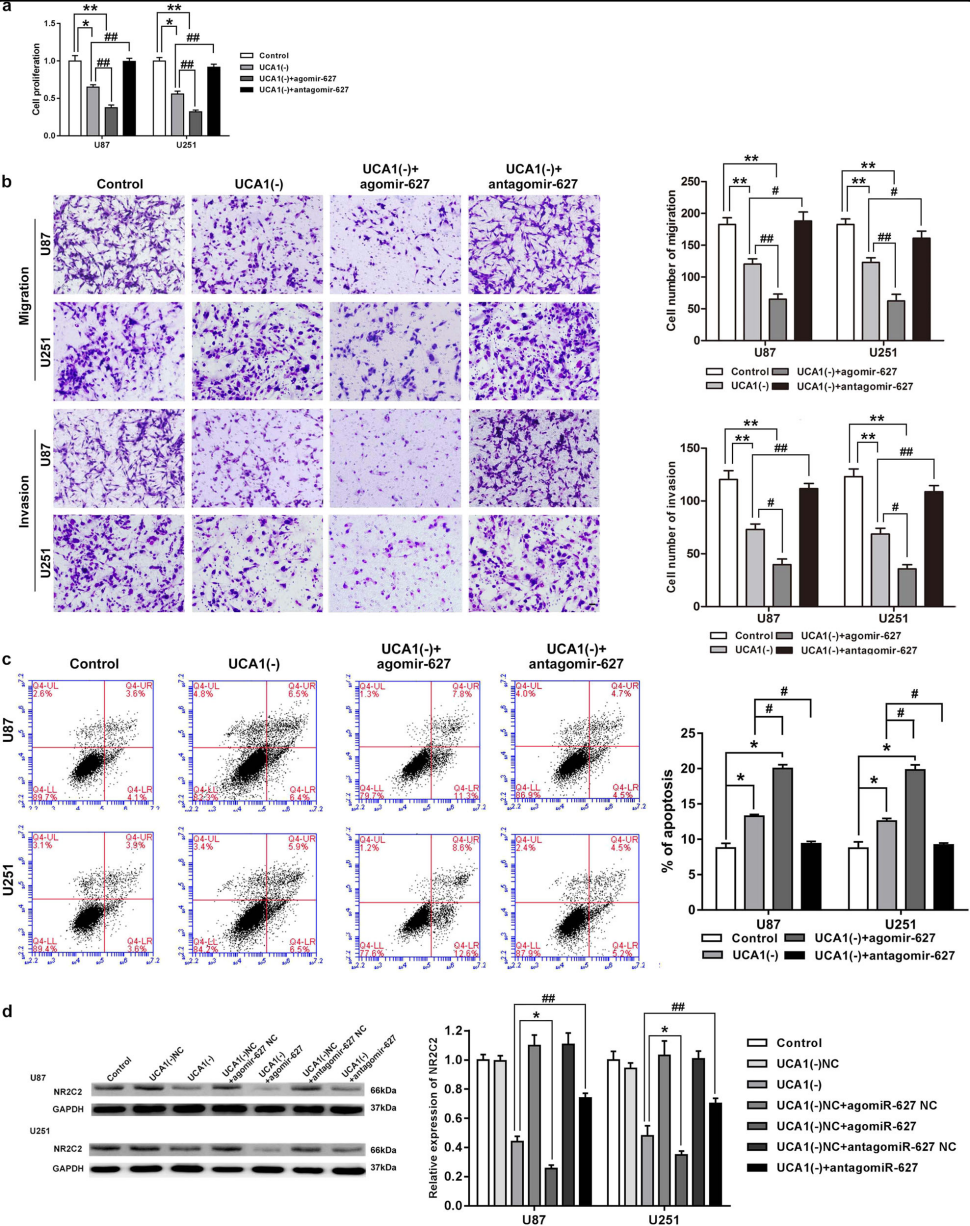
MiR-627-5p mediated the tumor-suppressive effects of UCA1 knockdown on glioma cell lines. **a** Cell Counting Kit-8 (CCK-8) assay was used to measure the effect of UCA1 and miR-627-5p on cell proliferation in U87 and U251 cells. **b** Transwell assay was used to evaluate the effect of UCA1 and miR-627-5p on cell migration and invasion of U87 and U251 cells. **c** Flow cytometry analysis was used to evaluate the effect of effect of UCA1 and miR-627-5p on cell apoptosis of U87 and U251 cells. Error bars represent as the mean ± SD (*n* = 3, each group). *P < 0.05, ^#^*P* < 0.05. Scale bars represent 20 μm. **d** Western blot analysis for Nuclear receptor subfamily 2 group C member 2 (NR2C2) expression level in U87 and U251 cells. Error bars represent as the mean ± SD (*n* = 3, each group). *P < 0.05, ^##^*P* < 0.01. Scalebars represent 20 μm

To clarify whether UCA1 was involved in the regulation of the influence of miR-627-5p had on the malignancies of glioma cells, cotransfection of miR-627-5p and UCA1 were assessed. As shown in Fig. 5a, b, UCA1 knockdown with miR-627-5p overexpression enhanced the reduction in proliferation, migration, and invasion caused by UCA1 knockdown alone (*P* < 0.05), while UCA1 knockdown with miR-627-5p inhibition partially rescued the reduction caused by UCA1 knockdown alone(*P* < 0.05). Figure 5c shows that compared with UCA1(−) group, cell apoptosis increased in UCA1(−)+agomiR-627-5p group(*P* < 0.05), but decreased in UCA1(−)+antagomiR-627-5p group (*P* < 0.05). These results confirmed that UCA1 knockdown functioned in glioma cells by overexpressing miR-627-5p. We next assessed whether UCA1 knockdown inhibited NR2C2 expression by evaluating miR-627-5p. Figure 5d shows that UCA1 knockdown impaired NR2C2 expressions significantly (*P* < 0.05). Moreover, cotransfection of UCA1 knockdown and miR-627-5p overexpression enhanced the inhibition effect caused by UCA1 knockdown alone (*P* < 0.05), while cotransfection of UCA1 knockdown and miR-627-5p inhibition rescued the suppression effect (*P* < 0.01).

### Tumor-suppressive effects of miR-627-5p was mediated by NR2C2

**Fig. 6:**
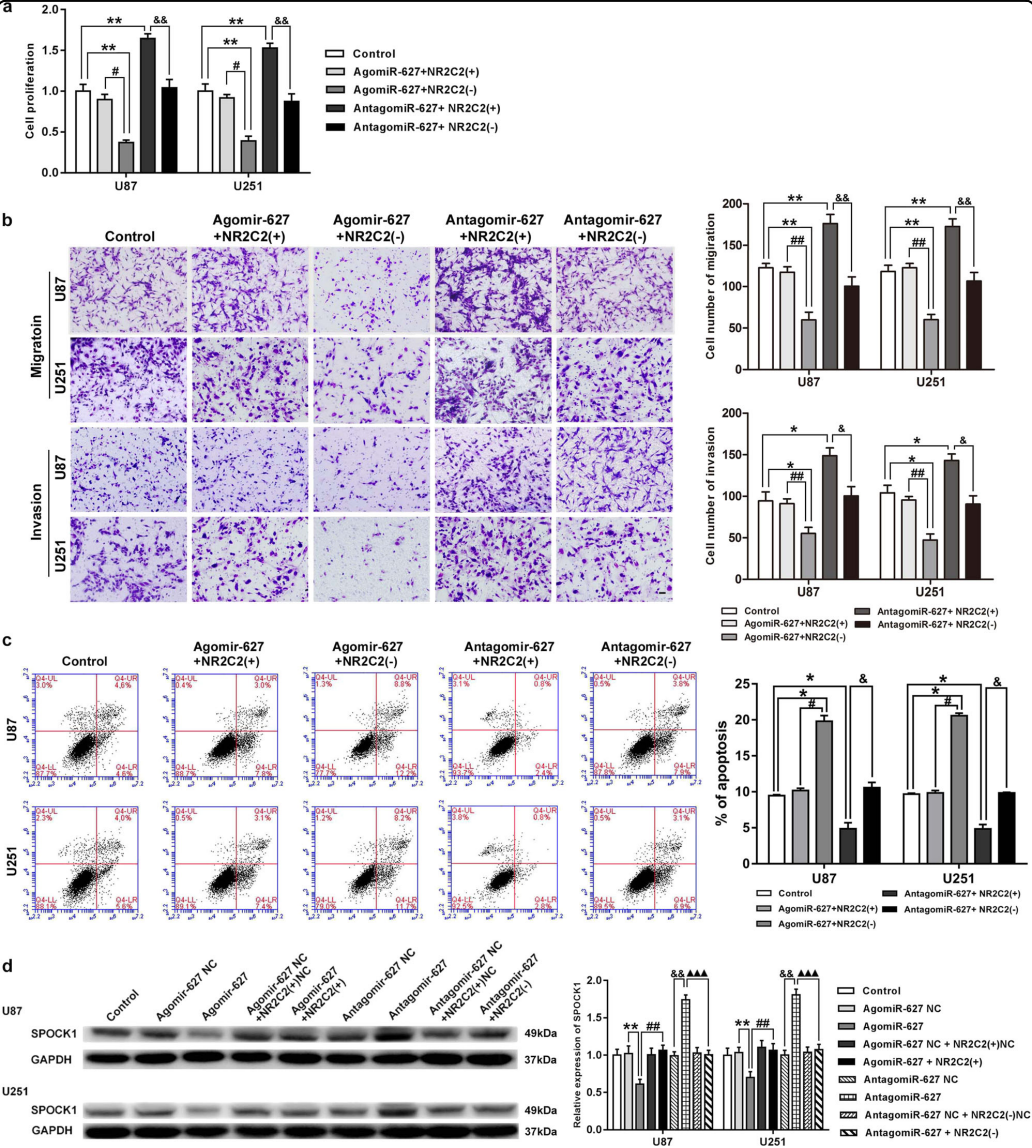
Interplay of NR2C2 and miR-627-5p. **a** Cell Counting Kit-8(CCK8) assay to evaluate the effect of miR-627-5p and NR2C2 on cell proliferation in U87 and U251 cells. **b** Transwell assay to evaluate the effect of miR-627-5p and NR2C2 on cell migration and invasion of U87 and U251 cells. **c** Flow cytometry analysis to evaluate the effect of miR-627-5p and NR2C2 on cell apoptosis of U87 and U251 cells. Error bars represent as the mean ± SD (*n* = 3, each group). *P < 0.05, ^#^*P* < 0.05, ^&^*P* < 0.05. Scale bars represent 20 μm. **d** Western blot analysis for SPARC/osteonectin, cwcv, and kazal-like domains proteoglycan 1 (SPOCK1) in U87 and U251 cells with the expression of miR-627-5p and NR2C2 changed. Error bars represent as the mean ± SD (*n* = 3, each group). **P < 0.01, ^##^*P* < 0.01, ^&&^*P* < 0.01, ^▲▲▲^*P* < 0.001

To clarify whether the influence of miR-627-5p had on glioma cells was mediated by NR2C2, we cotransfected U87 and U251 cells with miR-627-5p and NR2C2 constructs. Compared with the control group, proliferation, migration, and invasion of glioma cells decreased in agomiR-627-5p+NR2C2(−) group (*P* < 0.05), but increased in antagomiR-627-5p+NR2C2(+) group (Fig. 6a, b, *P* < 0.01). Moreover, cell apoptosis showed the opposite results (Fig. 6c, *P* < 0.05). These data suggested that miR-627-5p inhibited the malignant behaviors of glioma cells by down-regulating NR2C2. Western blot assay was conducted to further confirm that miR-627-5p inhibited SPOCK1 expression by down-regulating NR2C2. Figure 6d shows that compared with NC group, SPOCK1 expression decreased in agomiR-627-5p group (*P* < 0.01), but increased in antagomiR-627-5p group (*P* < 0.01). We also found that compared with agomiR-627-5p group, SPOCK1 expression significantly increased in agomiR-627-5p+NR2C2(+) group (*P* < 0.01), while compared with antagomiR-627-5p group, SPOCK1 expression significantly decreased in antagomiR-627-5p+NR2C2(−) group (*P* < 0.001).

### NR2C2 promoted SPOCK1 expression and bound to its promoters

**Fig. 7:**
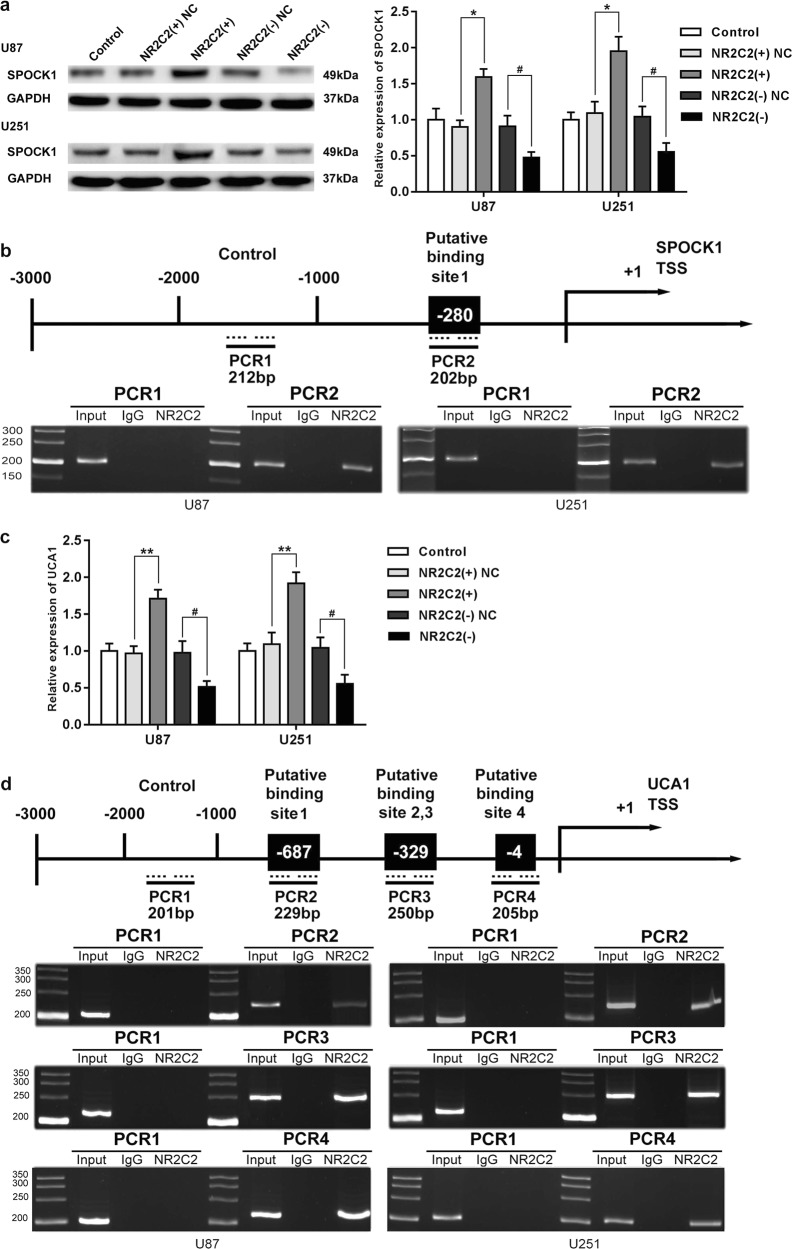
Identification of NR2C2-binding sites on SPOCK1 and UCA1 promoters. **a** Western blot analysis for SPOCK1 in U87 and U251 cells with the expression of NR2C2 changed. Error bars represent as the mean ± SD (*n* = 3, each group). **P < 0.01, ^#^*P* < 0.05. **b** NR2C2 bound to the promoter of SPOCK1 in U87 and U251 cell lines. Schematic representation of the human SPOCK1 promoter region 3000 bp upstream of the transcription start site (transcription start site (TSS), designated as +1). Polymerase chain reaction (PCR) was conducted with the resulting precipitated DNA. **c** Relative expression of UCA1 in U87 and U251 cells with the expression of NR2C2 changed. Error bars represent as the mean ± SD (*n* = 3, each group). **P < 0.01, ^#^*P* < 0.05. **d** NR2C2 bound to the promoter of UCA1 in U87 and U251 cell lines. Schematic representation of the human UCA1 promoter region 3000 bp upstream of the transcription start site (TSS, designated as +1). Polymerase chain reaction (PCR) was conducted with the resulting precipitated DNA

SPOCK1 was predicted as a direct target of NR2C2 by bioinformatics databases JASPAR. Figure 7a shows that compared with NC group, SPOCK1 expression was promoted in the NR2C2 (+) group (*P* < 0.05), but was inhibited in the NR2C2 (−) group (*P* < 0.05). TTS, transcription start site, of SPOCK1 was predicted using bioinformatics databases DBTSS HOME and a predicted NR2C2-binding site at −280 in SPOCK1 TSS was observed. Chromatin immunoprecipitation (ChIP) assay was conducted to investigate whether NR2C2 bound to SPOCK1 promoter directly. As shown in Fig. 7b, there was an interaction between NR2C2 and the SPOCK1 putative-binding site at −280 but control region showed no interaction.

### NR2C2 bound to UCA1 promoter and formed a positive feedback loop

We then detected the expression of UCA1 after NR2C2 was silenced and overexpressed. Figure 7c shows that UCA1 expression significantly increased in NR2C2(+) group compared with NC group (*P* < 0.01), whereas compared with the NC group, NR2C2 expression decreased in the NR2C2(−) group(*P* < 0.01). UCA1 transcription start sites (TSS) were predicted using DBTSS HOME and we identified some putative NR2C2-binding sites within the TSS of UCA1 using a bioinformatic database (JASPAR). Primers are designed to bind to UCA1 at −4, −329, and −687, and a region upstream of the potential-binding sites of NR2C2 which did not bind to UCA1 was amplified by PCR as a control. As shown in Fig. 7d ChIP assay confirmed the interactions between NR2C2 and UCA1 putative-binding sites, but not with the control region.

### UCA1 knockdown combined with uORF overexpression and silencing NR2C2 suppressed tumor growth in a BALB/c nude mice model

**Fig. 8:**
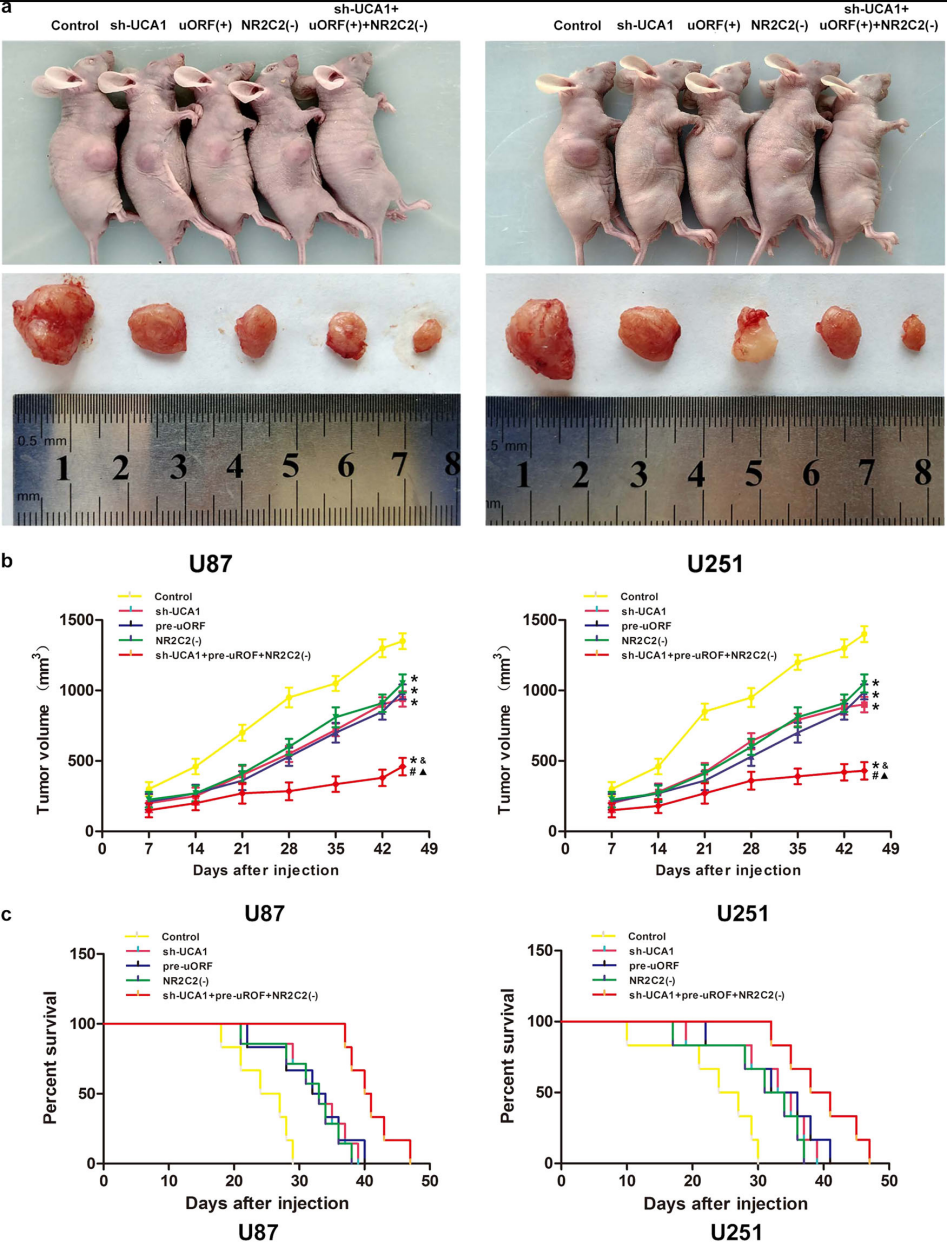
In vivo tumor xenografts study. **a **The stable expressing cells were used for the in vivo study. The nude mice carrying tumors from respective groups were shown. The sample tumor from respective group was shown. **b** Effect of UCA1 knockdown, NR2C2-uORF overexpression, NR2C2 knockdown, and combination of UCA1 knockdown, NR2C2-uORF overexpression, and NR2C2 knockdown on glioma xenograft volume and nude mice survival. Data represent mean ± SD (*n* = 7, each). *P < 0.05 vs. Control groups, ^#^*P* < 0.05 vs. NR2C2(−) groups, ^&^*P* < 0.05 vs. sh-UCA1 groups. ^▲^*P* < 0.05 vs. pre-uORF groups

To further confirm our results, in vivo tumor model was used. Results in Fig. 8a suggested that compared with Control group, sh-UCA1 group, pre-uORF group, NR2C2(−) group, and sh-UCA1+pre-uROF+NR2C2(−) group had smaller tumors (*P* < 0.05). But tumors in sh-UCA1+pre-uROF+NR2C2(−) group had the smallest volume compared with other groups (*P* < 0.05) and mice in sh-UCA1+pre-uROF+NR2C2(−) group had the longest survival time (Fig. 8b, *P* < 0.05).

## Discussion

In our study, we demonstrated that UCA1 expression was promoted in glioma tissues and cells. UCA1 knockdown led to an inhibition in proliferation, migration, and invasion, but a promotion in apoptosis in U87 and U251 cells. UCA1-regulated malignant behaviors of glioma cells by binding to its direct target miR-627-5p, which was confirmed to function as a tumor suppressor in glioma cells. UCA1 knockdown also inhibited the expression of transcription factor NR2C2. We also confirmed that NR2C2 was involved in regulating miR-627-5p-inducted inhibition of malignancies in U87 and U251 glioma cells. NR2C2 could promote activities of SPOCK1 and interacted with SPOCK1 promoters. Furthermore, NR2C2 also bound to UCA1 promoter and upregulated its expression, creating a positive feedback loop. In this process, NR2C2-uORF reduced the expression of NR2C2 mRNA thus decreasing NR2C2 protein expression and negatively regulated this feedback loop. In vivo study showed that UCA1 knockdown and NR2C2-uORF overexpression combined with silenced NR2C2 produced a tumor of the smallest volume and resulted in the longest survival time in nude mice.

LncRNAs impair protein expression by inhibiting RNA polymerase II activity^27^, interfering the cleavage of mRNAs or producing new siRNAs^28^, or change intracellular localization of proteins thus leading to its dysfuction^29^. LncRNAs are closely related to tumorigenesis and development^30^. Our present study found that UCA1 is overexpressed in glioma cells and tissues. UCA1 knockdown decreased cell proliferation, migration, and invasion but promoted apoptosis. These results implicated that UCA1 acted as an oncogene in glioma cells. Interestingly some studies showed similar results to our findings: UCA1 was upregulated in human osteosarcoma^31^ and promotes bladder cancer cell invasion and EMT^32^. UCA1 increased colorectal cancer cell proliferation and resistance to 5-fluorouracil^33^. And in vivo study also pointed out that compared with control group, silencing UCA1 produced a smaller tumor and resulted in a longer survival time in nude mice.

Our results confirmed that miR-627-5p expressed low in glioma tissues and cells. Furthermore, we found that overexpression of miR-627-5p resulted in an inhibition in cell proliferation, migration, and invasion but a promotion in apoptosis. These findings indicated that miR-627-5p negatively regulated the malignant behaviors of glioma cells. Just like our results, miR-627-5p is consistently reported to express at a low level in various human cancers. For example, vitamin D inhibited the expression of CYP3A4 by activating miR-627 and enhanced the therapeutic effect of irinotecan^34^. MiR-627 down-regulated BRCA2 expression and lowered the susceptibility of breast cancer^35^. MiR-627 mediated the epigenetic activities of vitamin D in colorectal cancer cells^36^. We defined miR-627-5p as a novel target of UCA1 and our results confirmed the significant interaction between UCA1 and miR-627-5p in the process of tumorigenesis, that is UCA1 knockdown impaired the malignant behaviors of glioma cells by promoting miR-627-5p expression.

Recent studies have shown that mRNAs, transcribed pseudogenes, lncRNAs, circRNAs may compete for the same miRNA response elements (MREs), thereby modulating miRNAs activity^37^. LncRNAs may function as competitive endogenous RNAs (ceRNAs) or molecular sponge to regulate miRNAs and its downstream targets^38^. For example, lnc-XIS regulates the expression of downstream FOXC1 and ZO-1 through binding to miR-137^39^. CRNDE acts as ceRNA and promoted GSCs proliferation, migration, invasion, and inhibit GSCs apoptosis by negatively regulating miR-186^40^. NEAT1 competitive binds to miR-129-5p and leads to inhibited papillary thyroid cancer progression^41^. In this study, we proved that UCA1 and NR2C2 mRNA-3'UTR shared a same MRE sequence (AGACUCA) to which miR-627-5p bound. MiR-627-5p overexpression together with UCA1 knockdown significantly impaired cell proliferation, migration, invasion, and promoted apoptosis but also impaired expression of NR2C2. Thus, UCA1 acted as a ceRNA to regulate miR-627-5p and affects downstream NR2C2 expression.

Our present study found that NR2C2 expression increased in glioma tissues and glioma cells. Overexpressed NR2C2 promoted proliferation, migration, and invasion of glioma cells but inhibited apoptosis. However, NR2C2 knockdown showed the opposite effects. These results suggested that NR2C2 was an oncogene in glioma cells. Similar results were as follows. NR2C2 regulated EZH2 through binding to its 5′UTR and promoted invasiveness of bladder cancer^42^. NR2C2 was upregulated in non-small cell lung cancer and was associated with poor prognosis of patients^43,44^. Silencing NR2C2 increased the resistance of hepatoma cells to cisplatin chemotherapy^45^. In vivo study also underlined that silencing NR2C2 inhibited tumor growth and prolonged life span in xenografted mouse model.

uORFs exist in most eukaryotic mRNAs extensively and function as regulatory elements in gene expression^46^. Studies have shown that uORFs regulate protein expression by inducing mRNA degradation^47,48^. In this study, we found an uORF in 5′UTR of NR2C2 mRNA variant 1 using the bioinfomatics database ORF Finder. This NR2C2-uORF expressed low in glioma tissue as well as in glioma cell lines and inhibited NR2C2 expression. Similarly, mutations in 5′UTR of Protein Kinase Mζ mRNA producing new uORFs may promote the expression of PKMζ mRNA and protein^49^, an uORF in CEBPA mRNA has been shown to act in cis as a translational repressor^50^, another uORF in Her-2 mRNA down-regulates its expression^51^.

We also clarified that miR-627-5p overexpression impaired NR2C2 expression. MiR-627-5p and NR2C2 co-overexpression signifcantly reversed the inhibition effect induced by transfection of agomiR-627-5p alone, revealing that miR-627-5p impaired malignancies of glioma cells by downregulating NR2C2 expression.

SPOCK1 belongs to the Ca^2+^-binding proteoglycan family which is involved in regulating adhesion, matrix cellular interactions, and cell growth^52^. We found that SPOCK1 was involved in NR2C2-induced regulation in glioma cells, whereas overexpression of NR2C2 increased the expression of SPOCK1. Furthermore, ChIP assay indicated that NR2C2 bound to SPOCK1 promoter and up-regulated SPOCK1 expression, thereby promoting malignant behaviors of glioma cells. Oncogene SPOCK1 is also seen in some other studies. For example, SPOCK1 has already been reported to promote glioma cell proliferation, migration, and invasion^53^. Silencing EPCR resulted in a reduction of SPOCK1 which reduced primary tumor growth of breast cancer^54^. SPOCK1 functioned as an oncogene through multiple signaling pathways, such as ERK/AKT pathways^55^ and mTOR-S6K pathways^56^.

In our study, we also confirmed that NR2C2 knockdown led to a decrease in UCA1 expression. ChIP assay indicated that NR2C2 bound to UCA1 promoter and upregulated UCA1 expression. Similar studies have reported that lncRNAs can be modulated by several key transcription factors. For example, MYC was involved in the proliferation and oncogenesis of colorectal tumor cells through targeting lncRNAs CDKN1A and CDKN2B^57^. E2F1 upregulated expression of lnc-GASL1 and led to decreased cell proliferation and in vivo tumor growth^58^. C-myc promoted proliferation and migration of pancreatic cancer cells by activating lnc-CCAT1^59^.

Based on the results above, we concluded that there is a positive feedback loop between NR2C2 and UCA1, in which inhibition of UCA1 decreased the NR2C2 expression through targeting miR-627-5p, whereas up-regulated NR2C2 promoted UCA1 expression, thus formed a positive feedback loop. Other reports that have shown some of slimilar results of this mechanism are as follows: H19 suppressed E-cadherin expression by promoting Slug expression and in return Slug can up-regulate H19 expression and activate its promoter. This may indicate the existence of a positive feedback regulation between H19 and Slug^60^.

Our study highlighted the importance of NR2C2-uORF in UCA1-miR-627-5p-NR2C2 feedback loop. This loop functioned mainly on modulating the oncogenetic effects of SPOCK1, which is a direct target as we have validated before. As a post-transcriptional regulatory element, NR2C2-uORF inhibited NR2C2 expression from a bypass through inhibiting NR2C2 mRNA expression thereby significantly inhibited the malignant behaviors of glioma cells. In vivo studies further confirmed that UCA1 knockdown combined with uORF overexpression and NR2C2 silenced suppressed tumor growth greatly and led to the longest survival in a xenograft mouse model. These results indicated that NR2C2-uORF acted as a tumor suppressor by directly targeting NR2C2 to inhibit the UCA1-miR-627-5p-NR2C2 feedback loop.

To conclude, this study for the first time demonstrated that UCA1 regulating the malignant behaviors of glioma cells by targeting transcription factor NR2C2 is mediated by miR-627-5p and eventually affects the expression of downstream protein SPOCK1, but NR2C2-47aa-uORF regulated the expression of NR2C2 directly to impair the effect of this feedback loop (Supplementary Fig. 1d). This study may provide a new strategy for the treatment of glioma.

## Electronic supplementary material

Supplymentary Figure 1

Supplymentary Figure 2

Supplementary figure legends

## References

[1] Khasraw, M., Ameratunga, M.S., Grant, R., Wheeler, H., Pavlakis, N. Antiangiogenic therapy for high-grade glioma. *Cochrane Database Syst. Rev.* **5**, CD008218 (2014). 10.1002/14651858.CD008218.pub3 25242542

[2] Van Meir EG (2010). Exciting new advances in neuro-oncology: the avenue to a cure for malignant glioma. CA.

[3] Nan Y (2017). Combinatorial therapy with adenoviral-mediated PTEN and a PI3K inhibitor suppresses malignant glioma cell growth in vitro and in vivo by regulating the PI3K/AKT signaling pathway. J. Cancer Res. Clin. Oncol..

[4] Kagami H, Akutsu T, Maegawa S, Hosokawa H, Nacher JC (2015). Determining associations between human diseases and non-coding RNAs with critical roles in NetworkControl. Sci. Rep..

[5] Schmitt AM, Chang HY (2016). Long noncoding RNAs in cancer pathways. Cancer Cell.

[6] Bartonicek N, Maag JL, Dinger ME (2016). Long noncoding RNAs in cancer: mechanisms of action and technological advancements. Mol. Cancer.

[7] Wang H (2017). LncRNA UCA1 in anti-cancer drug resistance. Oncotarget.

[8] Pan J (2016). Long non-coding RNA UCA1 promotes cisplatin/gemcitabine resistance through CREB modulating miR-196a-5p in bladder cancer cells. Cancer Lett..

[9] Yang YT (2016). Long non-coding RNA UCA1 contributes to the progression of oral squamous cell carcinoma by regulating the WNT/beta-catenin signaling pathway. Cancer Sci..

[10] Lou W (2017). MicroRNAs in cancer metastasis and angiogenesis. Oncotarget.

[11] Ling H (2016). Non-coding RNAs:. Adv. Exp. Med. Biol..

[12] Yu X, Zheng H, Chan MT, Wu WK (2017). Modulation of chemoresponsiveness to platinum-based agents by microRNAs in cancer. Am. J. Cancer Res..

[13] Banelli B (2017). MicroRNA in glioblastoma: an overview. Int. J. Genom..

[14] Padi SK, Zhang Q, Rustum YM, Morrison C, Guo B (2013). MicroRNA-627 mediates the epigenetic mechanisms of vitamin D to suppress proliferation of human colorectal cancer cells and growth of xenograft tumors in mice. Gastroenterology.

[15] Liu S (2014). Differential roles of PPARgamma vs TR4 in prostate cancer and metabolic diseases. Endocr.-Relat. Cancer.

[16] Qiu X (2015). TR4 nuclear receptor increases prostate cancer invasion via decreasing the miR-373-3p expression to alter TGFbetaR2/p-Smad3 signals. Oncotarget.

[17] Zhang L (2015). Testicular orphan receptor 4 (TR4) is a marker for metastasis and poor prognosis in non-small cell lung cancer that drives the EMT phenotype. Lung Cancer.

[18] Ding X (2015). TR4 nuclear receptor promotes prostate cancer metastasis via upregulation of CCL2/CCR2 signaling. Int. J. Cancer.

[19] Wethmar K, Smink JJ, Leutz A (2010). Upstream open reading frames: molecular switches in (patho)physiology. BioEssays.

[20] Cabrera-Quio LE, Herberg S, Pauli A (2016). Decoding sORF translation—from small proteins to gene regulation. RNA Biol..

[21] Barbosa C, Peixeiro I, Romao L (2013). Gene expression regulation by upstream open reading frames and human disease. PLoS Genet..

[22] Johnstone TG, Bazzini AA, Giraldez AJ (2016). Upstream ORFs are prevalent translational repressors in vertebrates. EMBO J..

[23] Sajjanar B (2017). Untranslated regions (UTRs) orchestrate translation reprogramming in cellular stress responses. J. Therm. Biol..

[24] Jin X, Turcott E, Englehardt S, Mize GJ, Morris DR (2003). The two upstream open reading frames of oncogene mdm2 have different translational regulatory properties. J. Biol. Chem..

[25] Koschmieder S (2007). CDDO induces granulocytic differentiation of myeloid leukemic blasts through translational up-regulation of p42 CCAAT enhancer binding protein alpha. Blood.

[26] Bisio A (2010). Functional analysis of CDKN2A/p16INK4a 5'-UTR variants predisposing to melanoma. Hum. Mol. Genet..

[27] Kotake Y (2016). Long non-coding RNA, PANDA, contributes to the stabilization of p53 tumor suppressor protein. Anticancer Res..

[28] Chatterjee D, Sanchez AM, Goldgur Y, Shuman S, Schwer B (2016). Transcription of lncRNA prt, clustered prt RNA sites for Mmi1 binding, and RNA polymerase II CTD phospho-sites govern the repression of pho1 gene expression under phosphate-replete conditions in fission yeast. RNA.

[29] Liu C (2017). Effects of LncRNA BC168687 siRNA on diabetic neuropathic pain mediated by P2X7 receptor on SGCs in DRG of rats. BioMed. Res. Int..

[30] Yang L (2018). LncRNAs regulate cancer metastasis via binding to functional proteins. Oncotarget.

[31] Li W, Xie P, Ruan WH (2016). Overexpression of lncRNA UCA1 promotes osteosarcoma progression and correlates with poor prognosis. J. Bone Oncol..

[32] Luo J (2017). LncRNA UCA1 promotes the invasion and EMT of bladder cancer cells by regulating the miR-143/HMGB1 pathway. Oncol. Lett..

[33] Bian Z (2016). LncRNA-UCA1 enhances cell proliferation and 5-fluorouracil resistance in colorectal cancer by inhibiting miR-204-5p. Sci. Rep..

[34] Sun M, Zhang Q, Yang X, Qian SY, Guo B (2016). Vitamin D enhances the efficacy of irinotecan through miR-627-mediated inhibition of intratumoral drug metabolism. Mol. Cancer Ther..

[35] Cao J (2016). rs15869 at miRNA binding site in BRCA2 is associated with breast cancer susceptibility. Med. Oncol..

[36] Hao B (2015). Bioinformatic analysis of microRNA expression in Parkinson's disease. Mol. Med. Rep..

[37] Giza DE, Vasilescu C, Calin GA (2014). MicroRNAs and ceRNAs: therapeutic implications of RNA networks. Expert Opin. Biol. Ther..

[38] Tay Y (2011). Coding-independent regulation of the tumor suppressor PTEN by competing endogenous mRNAs. Cell.

[39] Yoshida M (2015). The transcription factor Foxc1 is necessary for Ihh-Gli2-regulated endochondral ossification. Nat. Commun..

[40] Yu H (2017). Knockdown of long non-coding RNA XIST increases blood-tumor barrier permeability and inhibits glioma angiogenesis by targeting miR-137. Oncogenesis.

[41] Zheng J (2015). CRNDE affects the malignant biological characteristics of human glioma stem cells by negatively regulating miR-186. Oncotarget.

[42] Zhu J (2015). TR4 nuclear receptor alters the prostate cancer CD133+ stem/progenitor cell invasion via modulating the EZH2-related metastasis gene expression. Mol. Cancer Ther..

[43] Fang F (2013). Testicular orphan nuclear receptor 4-associated protein 16 promotes non-small cell lung carcinoma by activating estrogen receptor beta and blocking testicular orphan nuclear receptor 2. Oncol. Rep..

[44] Lin SJ (2017). TR2 and TR4 orphan nuclear receptors: an overview. Curr. Top. Dev. Biol..

[45] Shen J (2016). TR4 nuclear receptor enhances the cisplatin chemo-sensitivity via altering the ATF3 expression to better suppress HCC cell growth. Oncotarget.

[46] Ye Y (2015). Analysis of human upstream open reading frames and impact on gene expression. Hum. Genet..

[47] Tanaka M (2016). The minimum open reading frame, AUG-stop, induces boron-dependent ribosome stalling and mRNA degradation. Plant Cell.

[48] Muller, C., et al. Reduced expression of C/EBPbeta-LIP extends health- and lifespan in mice. *eLife* **7**, 1102–1120 (2018). 10.7554/eLife.34985 PMC5986274 29708496

[49] Bal NV (2016). Upstream open reading frames located in the leader of protein kinase mzeta mRNA regulate its translation. Front. Mol. Neurosci..

[50] Perrotti D (2002). BCR-ABL suppresses C/EBPalpha expression through inhibitory action of hnRNP E2. Nat. Genet..

[51] Mehta A, Trotta CR, Peltz SW (2006). Derepression of the Her-2 uORF is mediated by a novel post-transcriptional control mechanism in cancer cells. Genes Dev..

[52] Fan LC, Jeng YM, Lu YT, Lien HC (2016). SPOCK1 is a novel transforming growth factor-beta-induced myoepithelial marker that enhances invasion and correlates with poor prognosis in breast cancer. PloS One.

[53] Yang J (2016). SPOCK1 promotes the proliferation, migration and invasion of glioma cells through PI3K/AKT and Wnt/beta-catenin signaling pathways. Oncol. Rep..

[54] Perurena N (2017). EPCR promotes breast cancer progression by altering SPOCK1/testican 1-mediated 3D growth. J. Hematol. Oncol..

[55] Zhang LQ, Wang Y, Zhang L (2015). Effects of shRNA-mediated knockdown of SPOCK1 on ovarian cancer growth and metastasis. Cell. Mol. Biol..

[56] Wang Y, Wang W, Qiu E (2017). SPOCK1 promotes the growth of Osteosarcoma cells through mTOR-S6K signaling pathway. Biomed. Pharmacother..

[57] Kim, T., et al. Role of MYC-regulated long noncoding RNAs in cell cycle regulation and tumorigenesis. *J. Natl. Cancer Inst.* **107**, 881–890 (2015). 10.1093/jnci/dju505 PMC4402359 25663692

[58] Gasri-Plotnitsky L (2017). A novel lncRNA, GASL1, inhibits cell proliferation and restricts E2F1 activity. Oncotarget.

[59] Yu Q (2016). Long non-coding RNA CCAT1 that can be activated by c-Myc promotes pancreatic cancer cell proliferation and migration. Am. J. Transl. Res..

[60] Matouk IJ (2014). Oncofetal H19 RNA promotes tumor metastasis. Biochim. Biophys. Acta.

