# Supplementary figures and images for: NR2C2-uORF targeting UCA1-miR-627-5p-NR2C2 feedback loop to regulate the malignant behaviors of glioma cells
Source: Cell Death Dis. 2018 Dec 5;9(12):1165. doi: 10.1038/s41419-018-1149-x (PMC6281640; doi:10.1038/s41419-018-1149-x)

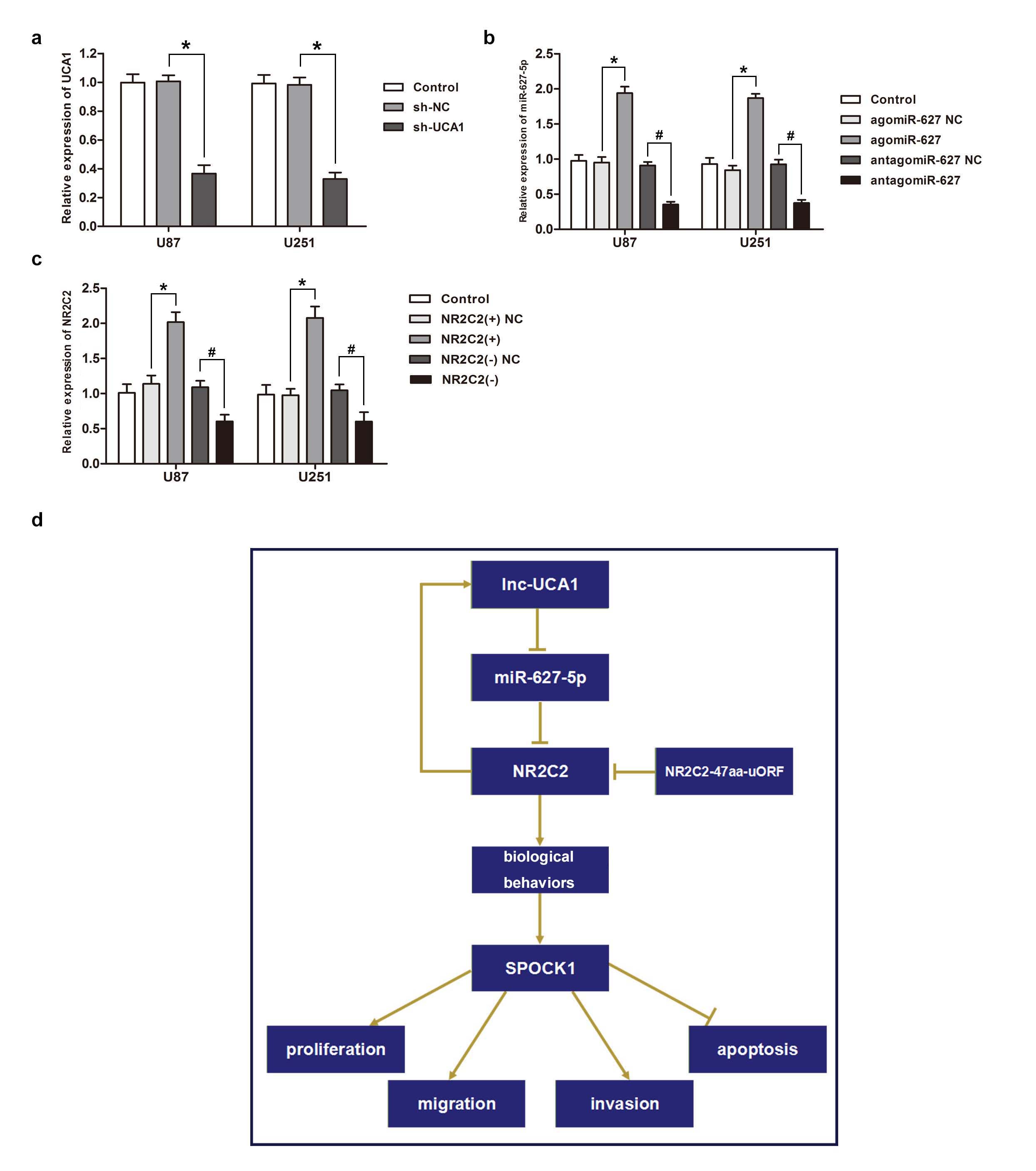

Supplement: Supplementary file 1 — Supplymentary Figure 1 [file 41419_2018_1149_MOESM1_ESM.jpg]

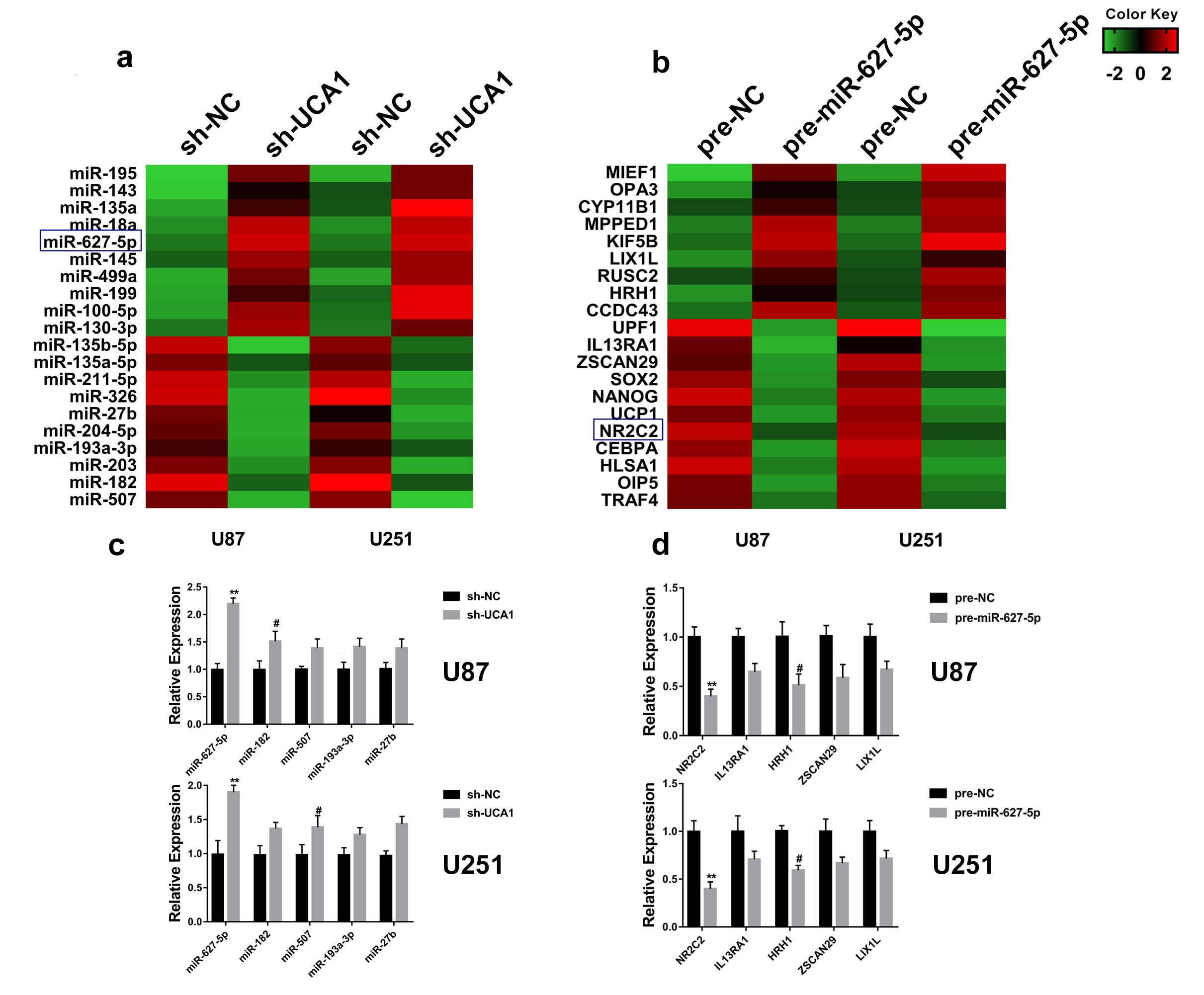

Supplement: Supplementary file 2 — Supplymentary Figure 2 [file 41419_2018_1149_MOESM2_ESM.jpg]
