# Supplementary material for: NR2C2-uORF targeting UCA1-miR-627-5p-NR2C2 feedback loop to regulate the malignant behaviors of glioma cells
Source: Cell Death Dis. 2018 Dec 5;9(12):1165. doi: 10.1038/s41419-018-1149-x (PMC6281640; doi:10.1038/s41419-018-1149-x)
Supplement: Supplementary file 3 — Supplementary figure legends [file 41419_2018_1149_MOESM3_ESM.doc]

**supplementary figure legends**

**Supplementary Figure 1**

**(a)** Relative UCA1 expression after U87 and U251 cells were stable transfected with sh-UCA1, ******P* <0.05. **(b)**Relative miR-627-5p expression after cells transfected with miR-627-5p mimics and miR-627-5p inhibitors, ******P* <0.05, ^#^*P* <0.05. **(c)**Relative NR2C2 expression after U87 and U251 cells were stable transfected with NR2C2 sh-NR2C2, ******P* <0.05, ^#^*P* <0.05. **(d)** The Schematic representation of the mechanism underlying the UCA1-miR-627-5p-NR2C2 feedback loop and the role of NR2C2-uORF in regulating this loop in glioma cells.

**Supplementary Figure 2. MiRNA and transcription factor microarrays data in U87, and U251 cells.
(a)** miRNA gene expression profiles as obtained from samples in three groups as indicated. **(b)** transcription factor gene expression profiles as obtained from samples in three groups as indicated. **(c)** qRT-PCR was performed to validate the selected miRNA. **(d)** qRT-PCR was performed to validate the selected transcription factor.
